# Impact of acute dynamic exercise and arterial shear rate modification on radial artery low-flow mediated constriction in young men

**DOI:** 10.1007/s00421-022-04963-x

**Published:** 2022-05-13

**Authors:** Mohammad H. Alali, Rebekah A. I. Lucas, Rehan T. Junejo, James P. Fisher

**Affiliations:** 1 School of Sport, Exercise and Rehabilitation Sciences, College of Life and Environmental Sciences, University of Birmingham, Birmingham, United Kingdom; 2 Department of Life Sciences, Faculty of Science and Engineering, Manchester Metropolitan University, Manchester, United Kingdom; 3 Department of Physiology, Faculty of Medical and Health Sciences, Manaaki Manawa-The Centre for Heart Research, University of Auckland, 85 Park Road, Grafton, Auckland, 1142 New Zealand

**Keywords:** Low-flow mediated constriction, Leg cycling, Shear rate, Vascular function

## Abstract

**Purpose:**

Leg cycling exercise acutely augments radial artery low-flow mediated constriction (L-FMC). Herein, we sought to determine whether this is associated with exercise-induced changes in arterial shear rate (SR).

**Methods:**

Ten healthy and recreationally active young men (23 ± 2 years) participated in 30 min of incremental leg cycling exercise (50, 100, 150 Watts). Trials were repeated with (Exercise + WC) and without (Exercise) the use of a wrist cuff (75 mmHg) placed distal to the radial artery to increase local retrograde SR while reducing mean and anterograde SR. Radial artery characteristics were measured throughout the trial, and L-FMC and flow mediated dilatation (FMD) were assessed before and acutely (~ 10 min) after leg cycling.

**Results:**

Exercise increased radial artery mean and anterograde SR, along with radial artery diameter, velocity, blood flow and conductance (*P* < 0.05). Exercise + WC attenuated the exercise-induced increase in mean and anterograde SR (*P* > 0.05) but also increased retrograde SR (*P* < 0.05). In addition, increases in radial artery blood flow and diameter were reduced during Exercise + WC (Exercise + WC vs. Exercise, *P* < 0.05). After Exercise, L-FMC was augmented (− 4.4 ± 1.4 vs. − 13.1 ± 1.6%, *P* < 0.05), compared to no change in L-FMC after Exercise + WC (− 5.2 ± 2.0 vs. − 3.0 ± 1.6%, *P* > 0.05). In contrast, no change in FMD was observed in either Exercise or Exercise + WC trials (*P *> 0.05).

**Conclusions:**

These findings indicate that increases in L-FMC following exercise are abolished by the prevention of increases radial artery diameter, mean and anterograde SR, and by elevation of retrograde SR, during exercise in young men.

## Introduction

The endothelium plays an essential role in maintaining vascular homeostasis (Birk et al. 2012; Green et al. 2017), while endothelial dysfunction is an early manifestation of atherosclerotic disease, arterial atheroma as well as other major cardiovascular diseases (Hambrecht et al. 2003; Watts et al. 2004). Flow mediated dilatation (FMD) is a non-invasive method widely used to assess endothelial function as it evokes an endothelial-dependent vasodilatory response to acute hyperemia (i.e., increased shear stress; mechanical interaction between the red blood cells and the blood vessel wall) in a conduit artery (e.g., brachial, femoral, radial) (Corretti et al. 2002). In contrast to FMD, acute reductions in conduit artery blood flow (e.g., decreased shear stress) induced by a period of distal ischemia, evokes a low-flow mediated constriction (L-FMC) (Gori et al. 2008; Humphreys et al. 2014). Like FMD, L-FMC is impaired in individuals with cardiovascular disease risk factors and is suggested to provide information that is complimentary to that provided by FMD (Gori et al. 2008, 2012). However, while the influence of acute environmental (e.g., heat, hypoxia) and physiological (e.g., exercise, mental stress) stimuli on FMD has been widely studied (Dawson et al. 2012; Vianna et al. 2014; Tremblay et al. 2019; Crandall and Wilson 2011), limited work has investigated how such interventions effect L-FMC and the underlying mechanisms (Alali et al. 2020).

Exercise training improves cardiovascular outcomes and more specifically enhances endothelial function (Watts et al. 2004; Fletcher et al. 2013). Such beneficial effects on the endothelium may be a result of exercise-induced increases in anterograde shear stress (Laughlin et al. 2008; Tinken et al. 2010; Birk et al. 2013). We have previously observed that L-FMC is enhanced immediately (~ 10 min) following a bout of acute dynamic exercise (Elliott et al. 2018), but it is not known whether this is attributable to an acute enhancement of endothelial function, secondary to changes in the pattern of shear stress (Dawson et al. 2012). Although we have previously observed that whole-body heating elevated radial artery shear rate, diameter, and blood flow but did not significantly change L-FMC (Alali et al. 2020). Notably, dynamic exercise with a large muscle mass acutely decreases the conduit artery FMD response for ~ 1 h, although this is not universally observed (Dawson et al. 2013; Elliott et al. 2018). This post-exercise attenuation of the FMD response may be a consequence of increased sympathetic vasoconstrictor tone (Atkinson et al. 2015; Thijssen et al. 2014; Padilla et al. 2010). Thus it is possible that enhanced L-FMC immediately (~ 10 min) following a bout of acute dynamic exercise (Elliott et al. 2018) is attributable to an increased sympathetic vasoconstrictor tone, rather than a change in the pattern of local blood flow.

Given this background, the aim of this investigation was to determine whether acute exercise-induced increases in L-FMC in young healthy individuals occurs because of a change in the pattern of blood flow that upregulates endothelial function. To achieve this, the influence of a bout of dynamic leg cycling exercise on radial artery blood flow pattern, FMD and L-FMC was investigated. Exercise trials were performed both with and without the experimental induction of a reduced mean SR and increase in retrograde SR in the radial artery by inflating a pneumatic cuff to 75 mmHg placed distal to the site of investigation (Carter et al. 2013; Thijssen et al. 2014, 2009). We hypothesized that wrist cuff inflation to reduce mean SR and augment retrograde SR during leg cycling exercise would attenuate radial artery L-FMC in young men. Investigations were focused on the post-exercise period because of its association with cardiovascular risk (e.g., coronary vasospasm/ischemia, cardiac arrhythmias) (Goodman et al. 2016; Akutsu et al. 2002; Albert et al. 2000).

## Methods

### Ethical approval

All study procedures were approved by the University of Birmingham, Science Technology Engineering and Mathematics Ethical Review Committee (approval number; ERN_18-0523). Written informed consent was obtained from all participants and studies conformed to the most recent revision of the Declaration of Helsinki, apart from registration in a database.

### Participant characteristics

Eleven healthy young men (age 23 ± 2 years; height: 175.5 ± 2.4 cm; weight; 71 ± 3 kg) were recruited to undertake three separate experimental trials. The following inclusion criteria, as assessed by pre-participation general health and physical activity questionnaires, were used for the participants: (1) normotensive (e.g., resting blood pressure < 140/90) and free from pulmonary, cardiovascular and metabolic disease; (2) engaging in regular exercise training (≥ 3 days per week) and no physical limitations; (3) non-smokers and medication free. Prior to experimental trials participants were requested to abide to the following guidance: no food or beverages ≥ 6 h, no alcohol or caffeine for ≥ 12 h, no polyphenol rich food/beverages for ≥ 18 h, no vigorous exercise for ≥ 48 h and no vitamin supplements for ≥ 72 h. Ten participants completed the experiment, and one participant withdrew from the study after the first trial for personal reasons.

### Experimental measures

Heart rate (HR) was assessed using a standard lead II surface electrocardiogram, and an automated sphygmomanometer was used to obtain systolic and diastolic blood pressure (BP) from the left brachial artery (Tango + , SunTech Medical Instruments, Raleigh, NC, USA). The right forearm was supported at heart level, while radial artery diameter and blood velocity were obtained using duplex Doppler ultrasound (Terason uSmart 3300, Teratech Corporation, Burlington, MA, USA). More specifically, the radial artery was imaged using a 4–15 MHz multi-frequency linear-array probe (Terason uSmart 15L4) that was held stable using and an adjustable holder and positioned 10–15 cm distal to the medial epicondyle. B-mode imaging and pulse-wave mode were used to gather radial artery diameter and peak blood velocity, respectively. Measurements were taken in line with recent technical recommendations (Thijssen et al. 2019). Automated edge detection and wall tracking algorithms software was used to record and save radial artery images (Cardiovascular Suite Version 3.4.1, FMD Studio, Pisa, Italy).

### Experimental protocol

Participants attended the laboratory on four separate occasions: one familiarisation session and three experimental trials. At the familiarisation session the equipment and the experimental procedures were demonstrated. Each experimental trial was carried out on a separate day with all three trials occurring within 14 days. All experimental trials were performed at the same time of day to avoid the potential confounding influences of diurnal variations in the measured variables (e.g., endothelial function) (Facer-Childs et al. 2019). Trials were conducted in temperature-controlled laboratory that was maintained at 23 °C. At each experimental session radial artery function (e.g., L-FMC and FMD) was assessed twice, both before and after either (1) 30 min of leg cycling exercise intervention (Exercise), (2) 30 min of leg cycling exercise intervention while a cuff placed around the right wrist was inflated to 75 mmHg to modify the blood flow and SR patterns of right radial artery (Exercise + WC), or (3) 30 min of quiet rest (Time Control). The order of the trials was randomised by the simple allocation concealment technique.

Each experimental trial commenced with the participants being seated on a repurposed car seat that was attached to a sturdy frame on which a cycle ergometer (Angio, Lode, Amsterdam, the Netherlands) was also mounted, and being instrumented for the measurement of HR, BP and radial artery hemodynamics. Participants remained in this upright seated position for the duration of each experimental trial. An inflatable cuff was placed around the right wrist and used for both the blood flow occlusion phase of the L-FMC and FMD assessment and to manipulate blood flow pattern during the Exercise + WC trial. Participants then rested for 20 min after which baseline measures of HR, BP and radial artery variables were obtained. Radial artery function (L-FMC, FMD) was then assessed. This consisted of baseline (1 min), wrist cuff inflation to ≥ 220 mmHg (5 min), and recovery/cuff deflation (3 min) periods. Following this, for the Exercise and Exercise + WC trial, participants then undertook a 30 min bout of leg cycling on a semi-recumbent cycle ergometer. The workload was initially set at 50 W and thereafter increased by 50 W every 10 min (50 W, 100 W and 150 W), during which cycling cadence was maintained at 60 rpm. Following this, participants recovered for 10 min and radial artery function was reassessed. During the Exercise + WC trial, the wrist cuff was inflated to 75 mmHg immediately following baseline and remained inflated throughout trial. In Time Control trial, the leg cycling was replaced with a quiet seated rest period and radial artery function assessed pre- and post-intervention as for Exercise and Exercise + WC.

### Data analysis

Mean arterial pressure (MAP) was calculated as: diastolic BP (mmHg) + [0.33 + (HR × 0.0012)] × [systolic BP (mmHg) − diastolic BP (mmHg)] (Razminia et al. 2004). Blood flow (ml/min) was calculated from radial artery diameter and mean blood velocity as: mean blood velocity (cm/s) × π × radius (cm)^2^ × 60. Vascular conductance was calculated as radial arterial blood flow (ml/min) divided by MAP (mmHg). Arterial wall shear rate (SR, s^−1^) was estimated, without consideration of the blood viscosity, as: 4 × mean blood velocity (cm/s) divided by diameter (cm). Anterograde and retrograde SR were calculated using positive and negative blood velocities, respectively.

Steady-state measurements of HR, BP and radial artery variables were recorded at baseline and every 5 min throughout the Exercise, Exercise + WC and Time Control trials. At each 5 min time point, the radial artery was assessed for 60 s and the highest quality continuous 20 s section then analyzed. L-FMC was calculated as the change from average baseline (1 min) diameter to the average diameter of the last 30 s of cuff occlusion period (Gori et al. 2008). FMD was determined from the change in baseline diameter to the maximal diameter observed in the post-occlusion period (Gori et al. 2008). Responses of L-FMC and FMD are presented as relative (%) and absolute (mm). Total vessel reactivity (TVR, %) was calculated using the following equation (Alali et al. 2020):

$$\frac{{{\text{FMD diameter }}\left( {{\text{mm}}} \right) - {\text{ LFMC diameter}}\left( {{\text{mm}}} \right)}}{{{\text{Baseline average diameter }}\left( {{\text{mm}}} \right)}} \times 100$$

The shear rate area under the curve (SR_auc_) and time to peak diameter was calculated from cuff release up to the point of maximum artery dilation. A simple ratio normalization of L-FMC against change in mean SR (difference between baseline SR and SR during L-FMC measurement period; L-FMC-to-∆mean SR ratio, au) and ratio of FMD against SR_auc_ (FMD-to-SR_auc_ ratio, au) were calculated to evaluate role of shear stress in mediating constriction and dilation (Padilla et al. 2008; Alali et al. 2020).

Recent guidelines suggest taking into consideration whether allometric scaling is required when analyzing FMD (Atkinson et al. 2013). Need for accurate scaling can be determined by obtaining the slope and the upper 95% confidence intervals (CI) of the relationship between natural log-transformed baseline and nadir/peak diameters (not to equal and less than a value of 1). Allometric scaling for baseline diameters was not conducted in current study as the slope of the relationship between log-transformed baseline and nadir/peak diameters were not significantly different from 1 (all slopes 0.93–0.79 and all upper 95% CI 1.81–0.98).

The percentage of age-predicted maximal heart rate (HR_max_) reached during each exercise workload (50, 100, 150 W) was calculated as; heart rate/HR_max_ × 100, where HR_max_ was calculated as 208–0.7 × age (Tanaka et al. 2001).

### Statistical analysis

Two-way repeated measures ANOVA were used to locate differences in hemodynamic responses, radial artery characteristics and blood flow with respect to time (baseline, 5, 10, 15, 20, 25, and 30 min), trial (Exercise, Exercise + WC, Time Control) and their interaction (time × trial). Two-way repeated measures ANOVA were also used to examine the main effects of time (Pre vs. Post), trial (Exercise, Exercise + WC, Time Control) and their interaction (time × trial), for radial artery characteristics and functional responses (e.g., L-FMC and FMD). Analysis of covariance ANCOVA was used to investigate the FMD response for the corrected shear rate stimulus (SR_AUC_-corrected-FMD%), using SR_AUC_ as a covariate (Thijssen et al. 2019). Significant main effects and interactions were investigated post hoc using Tukey’s tests. The level of significance for all tests at *P* < 0.05 was recognized as being statistically significant. All data are presented as mean (SD) unless stated otherwise. Repeated measures statistical analyses were performed using GraphPad Prism 8 (GraphPad Software, San Diego, CA) and ANCOVA analysis was performed with SPSS v. 21 (SPSS, Chicago, IL).

## Results

### HR and BP

**Fig. 1:**
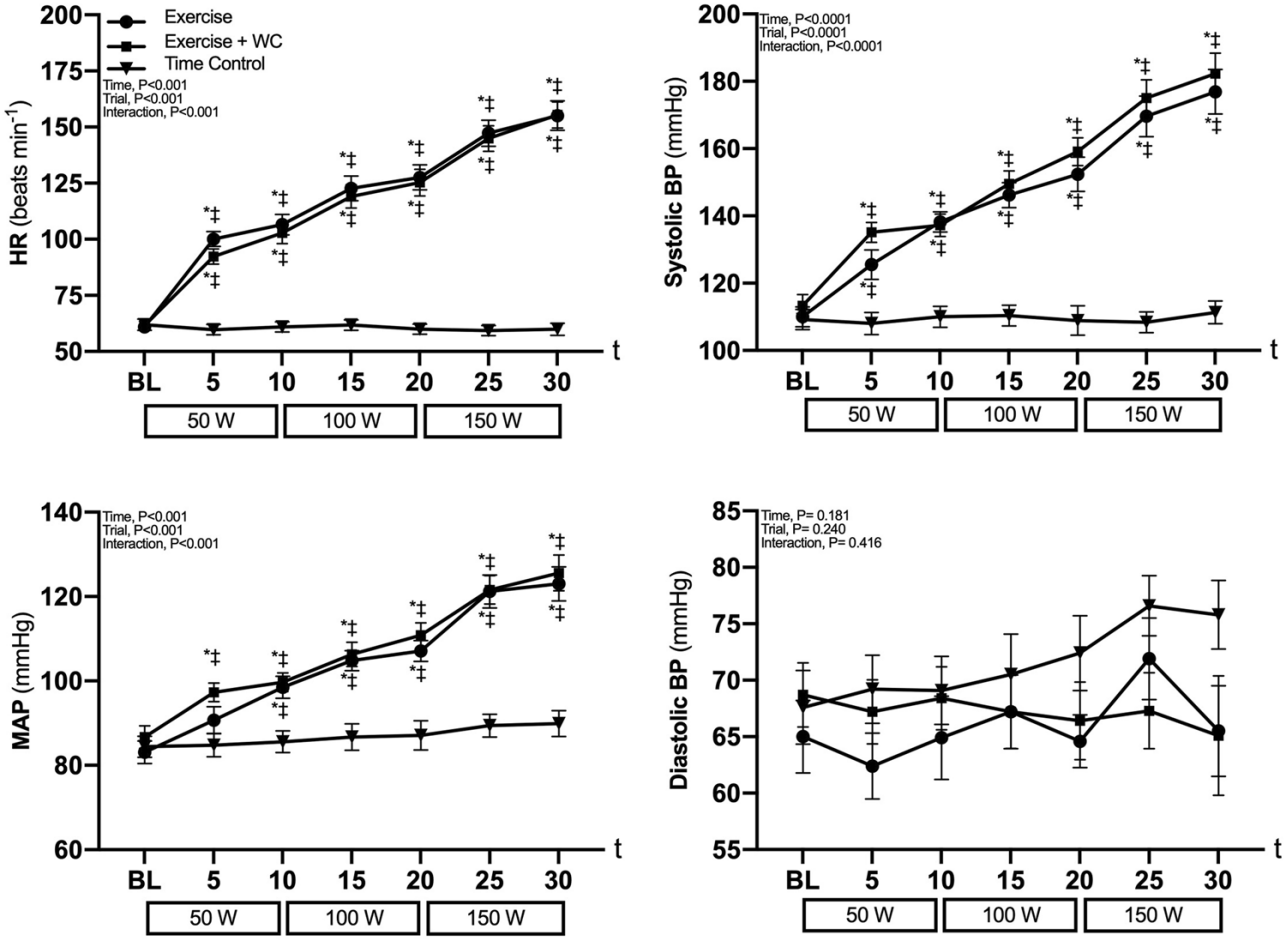
Cardiovascular responses heart rate (HR), systolic blood pressure (systolic BP), diastolic blood pressure (Diastolic BP) and mean arterial pressure (MAP) responses to leg cycling exercise (Exercise), leg cycling exercise with wrist cuff (Exercise + WC) and Time Control trials. Values are mean ± SE. *P < 0.05 vs. baseline (BL); †*P* < 0.05 vs. Exercise + WC; ‡*P* < 0.05 vs. Time Control

HR, systolic BP and MAP increased progressively (all, *P* < 0.05) and to a similar extent (all *P* < 0.05) from baseline in both the Exercise and Exercise + WC trials but were unchanged (*P* > 0.99) during the Time Control trial (Fig. 1). Diastolic BP did not differ from baseline in any trial (*P* ≥ 0.26). The 50-, 100- and 150 Watt workloads corresponded to 56 ± 8, 66 ± 9 and 81 ± 11%HR_max_ for Exercise trial and 54 ± 8, 65 ± 10 and 81 ± 10%HR_max_ for Exercise + WC trial, respectively.

### Radial artery characteristics

**Fig. 2:**
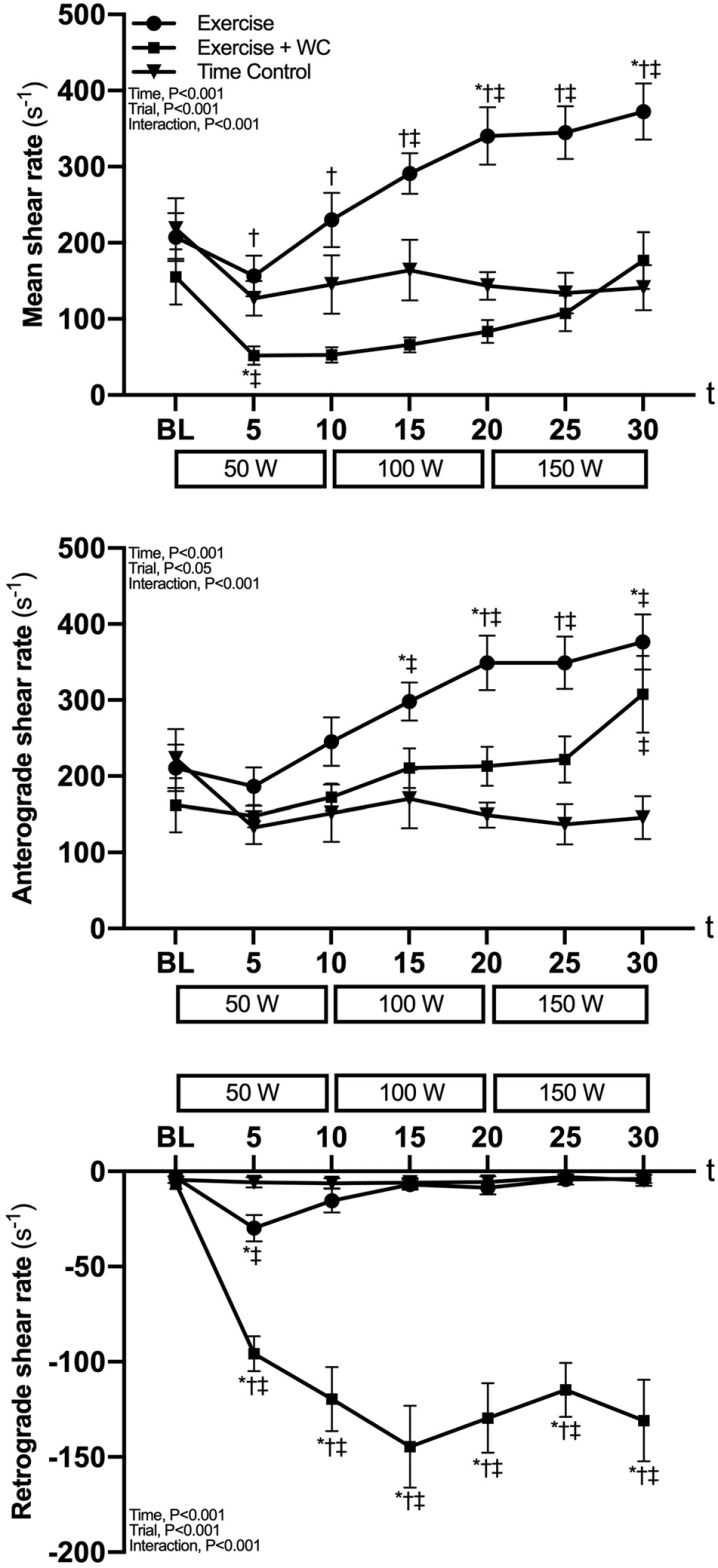
Radial artery blood flow pattern mean, anterograde and retrograde shear rate during leg cycling exercise (Exercise), leg cycling exercise with wrist cuff (Exercise + WC) and Time Control trials. Values are the mean ± SE. *P < 0.05 vs. baseline (BL); †*P* < 0.05 vs. Exercise + WC; ‡*P* < 0.05 vs. Time Control

Baseline mean, anterograde and retrograde SR did not differ between trials (Fig. 2). Mean and anterograde SR progressively increased during Exercise (*P* < 0.05). In contrast during Exercise + WC, mean SR initially decreased (*P* < 0.05, baseline vs 5 min) before returning to baseline (*P* ≥ 0.90, baseline vs. 25 and 30 min), and anterograde SR slightly increased (*P* < 0.05 vs. Time Control at 30 min). During Exercise, retrograde SR transiently increased (*P* < 0.05, baseline vs. 5 min) before returning to baseline (*P* ≥ 0.45, baseline vs. 10 min onwards), whereas during Exercise + WC retrograde SR was markedly increased (*P* < 0.01, baseline vs. 5 min onwards). In the Time Control trial, mean, anterograde and retrograde SR were unchanged (all, *P* ≥ 0.63) from baseline.

**Fig. 3:**
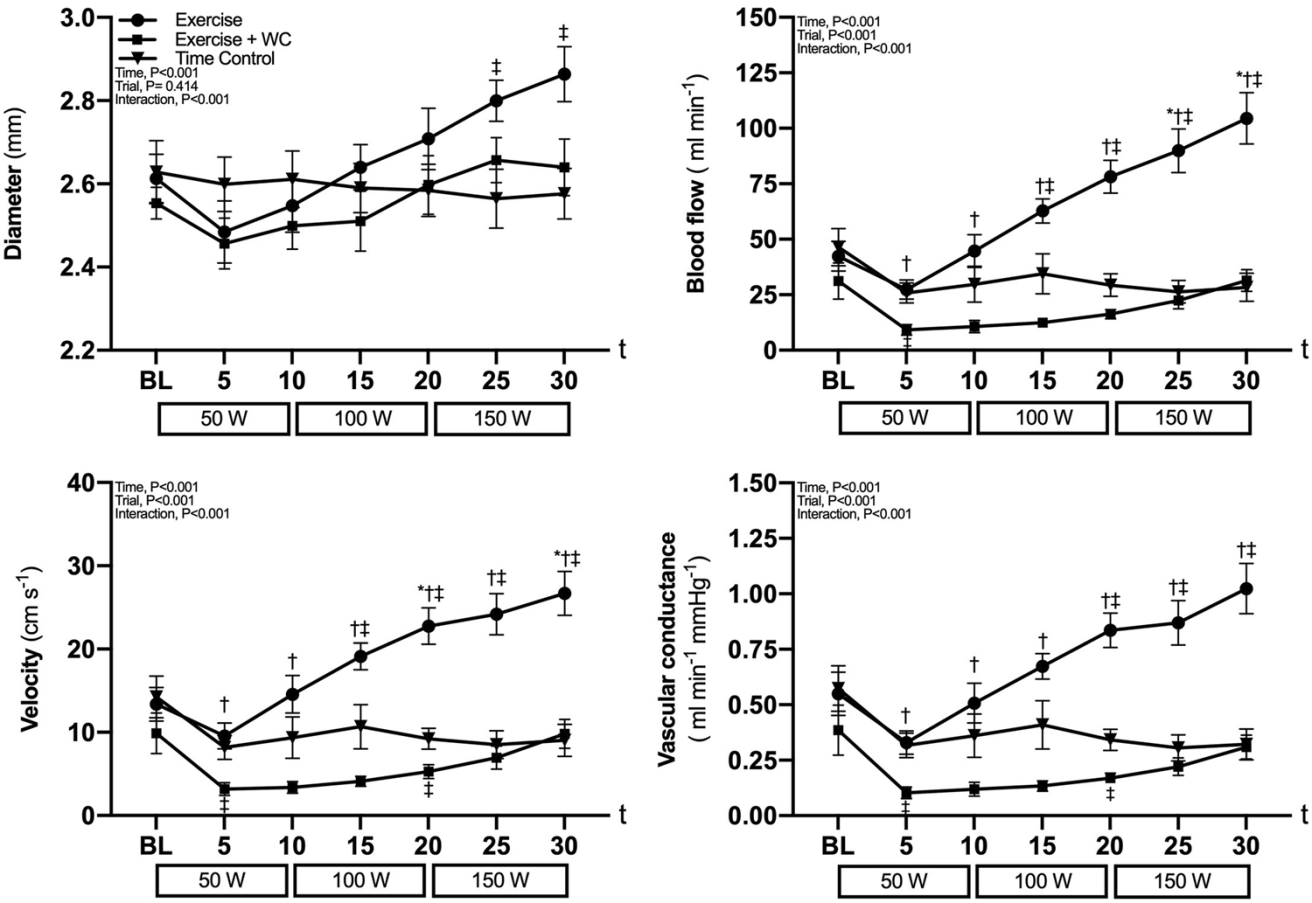
Radial artery characteristics radial artery blood flow, diameter, velocity and vascular conductance during leg cycling exercise (Exercise), leg cycling exercise with wrist cuff (Exercise + WC) and Time Control trials. Values are the mean ± SE. *P < 0.05 vs. baseline (BL); †*P* < 0.05 vs. Exercise + WC; ‡*P* < 0.05 vs. Time Control

Radial artery diameter, blood flow, velocity and vascular conductance were not different between all trials at baseline (all *P* ≥ 0.41, Fig. 3). During Exercise, radial artery diameter, blood flow, velocity and vascular conductance all progressively increased (all, *P* < 0.05 vs. Time Control at 25 and 30 min). In contrast, diameter, blood flow, velocity, and vascular conductance were not significantly different from baseline during Exercise + WC and Time Control (all *P* ≥ 0.06).

### Radial artery function responses

**Fig. 4:**
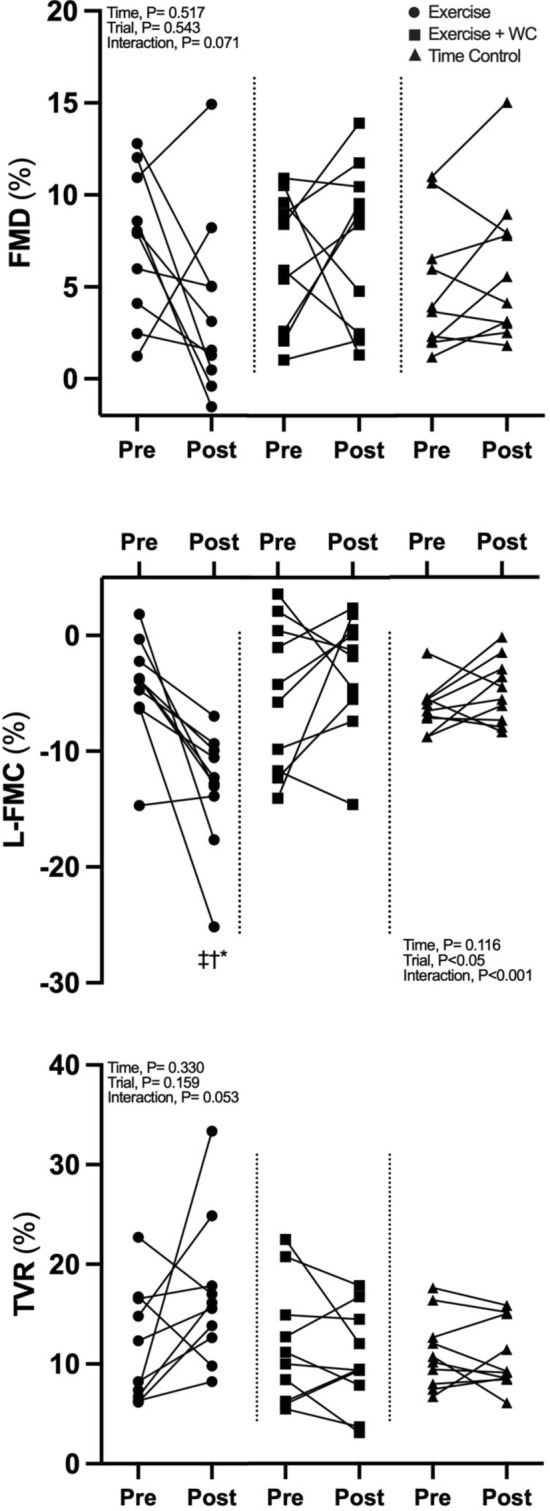
Radial artery function adial artery flow mediated dilatation (FMD), low-flow mediated constriction (L-FMC), and total vascular range (TVR) during leg cycling exercise (Exercise), leg cycling exercise with wrist cuff (Exercise + WC) and Time Control trials. Values are the mean ± SE. *P < 0.05 vs. baseline (BL); †*P* < 0.05 vs. Exercise + WC; ‡*P* < 0.05 vs. Time Control

Radial artery characteristics before and after the Time Control, Exercise and Exercise + WC trials are presented in (Table 1). FMD, L-FMC and TVR % responses were not different between trials at baseline (*P* ≥ 0.53, Fig. 4). The L-FMC % response increased significantly following Exercise (*P* < 0.05 vs Pre; *P* < 0.05 vs Exercise + WC; P < 0.05 vs Time Control). The time x Trial interaction for FMD (*P* = 0.074) and TVR (*P* = 0.053) failed to reach the threshold for statistical significance, however tendencies for a reduction in FMD and an increase in TVR was noted following the Exercise trial.

**Table 1:** Radial artery characteristics before [Pre] and after [Post] the Time Control, Exercise and Exercise + WC trials

	Time Control		Exercise		Exercise + WC		*P* values		
	Pre	Post	Pre	Post	Pre	Post	Trial	Time	Interaction
Baseline									
Diameter (mm)	2.63 (0.24)	2.57 (0.18)	2.61(0.18)	2.80(0.25)*‡	2.55(0.12)	2.66(0.18)	0.341	0.004	0.002
Velocity (cm/s)	14.25(7.95)	7.72(4.40)*	13.37 (6.40)	20.45(7.44)*†‡	9.89(7.70)	4.21(1.87)	< 0.001	0.253	0.001
Blood flow (ml/min)	46.45(26.35)	24.01(13.39)	42.35(21.21)	75.91(28.68)*†‡	31.20(25.75)	14.46(7.92)	< 0.001	0.733	< 0.001
Mean shear rate (s^−1^)	218.71(126.18)	121.55(70.84)	207.45(99.18)	295.16(115.24)†‡	155.34(114.68)	62.72(25.26) *	0.002	0.122	0.002
L-FMC									
Nadir diameter (mm)	2.46(0.22)	2.44(0.16)	2.50(0.23)	2.44(0.29)	2.42(0.20)	2.57(0.17)*	0.894	0.389	0.012
Δ Diameter (mm)	− 0.17(0.06)	− 0.13(0.07)	− 0.11(0.12)	− 0.36(0.13)*†‡	− 0.13(0.17)	− 0.09(0.14)	0.012	0.072	< 0.001
Mean shear rate (s^−1^)	18.41(7.14)	20.40(10.15)	22.13(5.21)	29.54 (9.03)*†‡	19.33(5.769)	19.20(6.913)	0.053	0.033	0.088
Δ Mean shear rate (s^−1^)	200.31(123.20)	101.15 (63.45)	185.32(98.82)	265.61(113.32)†‡	136.01(112.80)	43.53(24.75)*	0.002	0.093	0.002
L-FMC-to- Δ mean SR ratio (au)	− 0.043(0.032)	− 0.067(0.050)	− 0.009(0.052)	− 0.066(0.064)	− 0.050(0.113)	− 0.061(0.215)	0.892	0.117	0.592
FMD									
Peak diameter (mm)	2.75 (0.23)	2.72 (0.20)	2.81(0.24)	2.91(0.28)	2.72 (0.18)	2.85(0.14)*	0.426	0.028	0.065
Δ Diameter (mm)	0.12(0.09)	0.15(0.10)	0.20(0.10)	0.11(0.14)	0.17(0.10)	0.19(0.11)	0.595	0.549	0.085
Time to peak diameter (s)	101.10(51.810)	82.60(31.22)	85.50(44.47)	108.90(35.38)	97.50(46.84)	73.50(47.24)	0.717	0.559	0.164
SR_AUC_ (× 10^3^ s^−1^)	26.39 (23.31)	27.10(13.48)	17.50 (6.66)	37.50 (35.66)	17.20 (8.43)	27.61(20.29)	0.733	0.044	0.291
FMD-to-SR_AUC_ ratio (au)	0.35(0.37)	0.29(0.25)	0.47(0.31)	0.24(0.35)	0.51 (0.39)	0.43 (0.41)	0.388	0.194	0.728
SR_AUC_-corrected-FMD (%)	4.92(4.06)	6.01(4.06)	7.09(4.12)	4.24(4.18)	6.19(4.12)	7.41(4.06)	0.543	0.868	0.215

Values are means [SD]. L-FMC, low-flow mediated constriction; FMD, flow mediated dilatation; SR_AUC_, shear rate area under curve. *P* values represent 2-way repeated ANOVA results (Trial; Time Control, Exercise and Exercise + WC: Time; Pre and Post: Interaction, Trial x Time). *P* value for SR_AUC_-corrected-FMD (%) represent ANCOVA results

*P < 0.05 vs. Pre; †*P* < 0.05 vs. Exercise + WC; ‡*P* < 0.05 vs. Time Control

## Discussion

The primary objective of the present study was to determine whether augmented radial artery L-FMC following acute dynamic leg exercise is attributable to a change in local SR. Accordingly, the application of a cuff placed distal to radial artery (Exercise + WC) and inflated to 75 mmHg during leg cycling exercise was observed to attenuate exercise-induced increases in radial artery diameter, mean and anterograde SR, while significantly augmenting retrograde SR. In accordance with our hypothesis, this change in local SR prevented the exercise-induced increase in radial artery L-FMC in young men.

We have previously observed that leg cycling exercise acutely augments L-FMC in healthy adults (Elliott et al. 2018). The endothelium is one of the proposed mechanisms responsible for this L-FMC response to exercise (Dawson et al. 2012). Although, L-FMC is reportedly not altered by infusing NO synthase inhibitor (Gori et al. 2008), the inhibition of other endothelial derived vasodilatory substances (e.g., prostaglandins and endothelial-derived hyperpolarizing factors) does attenuate L-FMC, suggesting that it is partly endothelium mediated (Gori et al. 2008). Additional support for an endothelial contribution to radial artery L-FMC is provided by the observation that it is blunted by endothelial denudation induced by the radial artery catheterization procedure (Dawson et al. 2012). As observed by Elliot et al. (2018), and several others prior to this (Birk et al. 2012; Tinken et al. 2010), leg cycling exercise increases arm blood flow and anterograde SR, which is known to upregulate the release of endothelium-derived vasodilatory substances. This may explain the acutely increased radial L-FMC in the immediate post-exercise period (Elliott et al. 2018). Interestingly, acute whole-body heat stress was also observed to increase radial artery diameter, blood flow and anterograde SR during, however, no significant change in L-FMC was noted (Alali et al. 2020). However, there are important differences in the hemodynamic, thermoregulatory, and cellular signalling responses to acute exercise and whole-body heating (Cullen et al. 2020) that may explain the divergent L-FMC results.

To determine the endothelial contribution to the augmented post-exercise L-FMC response, we examined the effect of radial artery SR manipulation during leg cycling exercise. As expected, the application of a cuff inflated to 75 mmHg and placed distal to the radial artery prevented normal increases in mean and anterograde SR, whereas retrograde SR was observed to be markedly augmented. This is a well-recognized approach to modify local SR patterns during experimental manipulations (Thijssen et al. 2009; Johnson et al. 2012; Tinken et al. 2009). In vivo and in vitro studies of endothelial function have shown that this type of oscillatory shear stress, characterized by a high level of retrograde SR, causes endothelial dysfunction (e.g., increases proatherogenic genes, decreases FMD response) (Thijssen et al. 2009; Ziegler et al. 1998). At a cellular level, an increase in retrograde SR markedly reduces endothelial NO synthase (Ziegler et al. 1998). Given this, the reduction in L-FMC caused by Exercise + WC might be explained by a reduction in endothelial function due to the attenuated anterograde and mean SR, and/or the increased retrograde SR. However, other factors, including secondary changes in the prevailing arterial diameter, should also be considered.

Notably in the Exercise trial, the post-Exercise radial artery diameter significantly increased from pre-Exercise (pre 2.61 ± 0.18 mm vs. post: 2.80 ± 0.25 mm), while the reduction in shear rate during L-FMC assessment was augmented (pre ∆185.32 ± 98.82 s^−1^ vs. post ∆265.61 ± 113.32 s^−1^). Conversely, in the Exercise + WC trial radial artery diameter did not change pre—to—post exercise and in fact the change in mean shear rate was much lower post-exercise (∆43.53 ± 24.75 s^−1^) as compared to pre (∆136.01 ± 112.80 s^−1^). As such, the L-FMC-to-Δ mean SR ratio was not different among trials. This indicates that differences in the prevailing radial artery diameter at the time of L-FMC and the change in shear rate caused by the occlusion cuff inflation used in the assessment of L-FMC may also be important contributing factors to the responses observed.

The beneficial effects of exercise on FMD appear to be mediated by the associated periodic increases in conduit artery blood flow (Watts et al. 2004; Fletcher et al. 2013) and more specifically the increase in anterograde SR that is known to upregulate endothelial NO synthase expression (Laughlin et al. 2008). Several studies have observed that FMD is acutely attenuated in the post-Exercise period (Dawson et al. 2013; Hwang et al. 2012). Importantly, Atkinson et al. (Atkinson et al. 2015) showed that the attenuation of FMD following acute high-intensity dynamic exercise was prevented by administration of an oral alpha-adrenergic antagonist. This suggests that increases in sympathetic nerve activity cause the acutely reduced FMD post-Exercise, a conclusion supported by earlier observations that other acute sympatho-excitatory manoeuvres are also associated with reduced FMD (Hijmering et al. 2002). In agreement with Elliot et al. (Elliott et al. 2018), who used the same incremental exercise protocol as the present study, no significant reduction in FMD was noted following Exercise. However, there was a tendency for a significant interaction between time and trial (*P* = 0.071) and a numerically lower FMD post-Exercise (3.8 ± %) versus pre-Exercise (7.4 ± 4%, Fig. 4). Interestingly, FMD was not different following Exercise + WC despite the wrist cuff manipulation significantly augmented retrograde SR. Thijssen et al. (Thijssen et al. 2009) previously reported that brachial artery FMD was attenuated by increased retrograde SR induced by cuff inflation (e.g., 25, 50, 75 mmHg), while anterograde SR was unchanged. In addition, Johnson et al. (Johnson et al. 2012) reported that a wrist cuff (inflated to 60 mmHg) increased retrograde SR and attenuated brachial FMD following supine leg cycling exercise (90 W, 20 min). A potential explanation for the conflicting findings of these studies (Thijssen et al. 2009; Johnson et al. 2012) and those of the present study, may be that we observed a small but significant increase in anterograde SR during Exercise + WC (at 30 min time point) that may have counteracted the negative effects of retrograde SR on endothelial function. Nevertheless, the observed failure of Exercise + WC to diminish FMD in our study does not support the view that the attenuated L-FMC response at this time is attributable to a reduction in endothelial function.

### Methodological considerations

Our investigations were confined to an assessment of the radial artery and additional studies are needed to validate these results in other conduit arteries. We acknowledge that the brachial artery is frequently assessed in human studies of peripheral vascular function, although it is not uncommon for the radial artery to be interrogated. Furthermore, the radial artery is used as a graft in coronary bypass surgeries (Song et al. 2012), where it may be susceptible to functional vasospasm (He and Taggart 2016), further supporting the relevance of its study. Importantly, we observed radial artery blood flow, diameter and SR patterns during Exercise that are comparable to those previously documented for the brachial artery (Padilla et al. 2011; Birk et al. 2013). The L-FMC response is reported to be more commonly observed in radial artery than brachial artery (Weissgerber et al. 2010). Additional mechanistic insights would have been provided had a measurement of sympathetic nervous system activation been included in the present study. We can only assume that it was equivalent in the Exercise and Exercise + WC conditions, but in support of this neither HR nor BP were not different between the Exercise and Exercise + WC conditions. In the present study, only healthy young men were recruited which is a limitation. Both sex- and ovarian hormone concentrations can modify vascular function (Hashimoto et al. 1995). Regrettably, in the current study we were not sufficiently resourced to study young women at a standard phase (e.g., early follicular stage) or multiple phases of their menstrual cycle for the three separate trials. The present results cannot be extrapolated to women and further studies are needed to examine whether the findings of the present study are applicable to women. The exercise mode employed was incremental dynamic exercise (e.g., 50, 100, 150 Watts), which was well tolerated by participants and permitted the simultaneous acquisition of high-quality radial artery images. In addition, the leg cycling exercise modality and workloads employed are representative of those suggested for establishing and preserving cardiorespiratory fitness.

Assessments of radial artery function (i.e., L-FMC) were made at rest and immediately (~ 10 min) following acute dynamic exercise. The broader significance of this time period is that it is associated with an increased cardiovascular risk (e.g., coronary vasospasm/ischemia, cardiac arrhythmias) (Franklin et al. 2020; Thompson et al. 2007; Akutsu et al. 2002; Albert et al. 2000). There are known parallels between the vasodilatory responses of the peripheral conduit and coronary arteries (Anderson et al. 1995; Takase et al. 1998). As such our findings from the radial artery support the concept (Carter et al. 2013; Thijssen et al. 2014, 2009) that in healthy individuals increases mean and anterograde SR facilitate local vasodilatation, hyperaemia and functional capacity, thus making an important contribution to oxygen delivery via the coronary vasculature. Several studies have reported a temporal variation in FMD following exercise (Birk et al. 2013), but unfortunately only a single time point was assessed in this study and therefore further investigation are required to determine the time course of the L-FMC response post-exercise.

## Conclusions

These findings confirm that dynamic leg cycling exercise acutely increases radial artery mean and anterograde SR, leading to radial artery vasodilatation and an augmented L-FMC in young men. The application of a cuff placed distal to the radial artery and inflated during leg cycling exercise attenuated exercise-induced increases in mean and anterograde SR, significantly augmented retrograde SR, and prevented the vasodilatation of the radial artery during exercise. Importantly, these hemodynamic alterations acutely attenuated L-FMC. Collectively, these observations suggest that SR modifications explain the radial artery responses to acute leg cycling and the ensuing augmentation of L-FMC. Such findings are consistent with the view that lower limb exercise evokes a pattern of blood flow through the upper limb conduit arteries that modifies endothelial function such that local vasodilatation, hyperaemia and functional capacity (in terms of L-FMC) are acutely augmented.

## Abbreviations

BP: Blood pressure
ECG: Electrocardiograph
FMD: Flow mediated dilatation
HR: Heart rate
L-FMC: Low-flow mediated constriction
MAP: Mean arterial pressure
NO: Nitric oxide
SR: Shear rate
SR_AUC_: Shear rate area under the curve
TVR: Total vessel reactivity

## References

[CR1] Akutsu Y, Shinozuka A, Nishimura H, Li HL, Huang TY, Yamanaka H, Takenaka H, Munechika H, Katagiri T (2002). Significance of ST-segment morphology noted on electrocardiography during the recovery phase after exercise in patients with ischemic heart disease as analyzed with simultaneous dual-isotope single photon emission tomography. Am Heart J.

[CR2] Alali MH, Vianna LC, Lucas RAI, Junejo RT, Fisher JP (2020). Impact of whole body passive heat stress and arterial shear rate modification on radial artery function in young men. J Appl Physiol.

[CR3] Albert CM, Mittleman MA, Chae CU, Lee IM, Hennekens CH, Manson JE (2000). Triggering of sudden death from cardiac causes by vigorous exertion. N Engl J Med.

[CR4] Anderson TJ, Uehata A, Gerhard MD, Meredith IT, Knab S, Delagrange D, Lieberman EH, Ganz P, Creager MA, Yeung AC et al (1995) Close relation of endothelial function in the human coronary and peripheral circulations. J Am Coll Cardiol 26:1235–1241. 10.1016/0735-1097(95)00327-4 10.1016/0735-1097(95)00327-4 7594037

[CR5] Atkinson G, Batterham AM, Thijssen DH, Green DJ (2013). A new approach to improve the specificity of flow-mediated dilation for indicating endothelial function in cardiovascular research. J Hypertens.

[CR6] Atkinson CL, Lewis NC, Carter HH, Thijssen DH, Ainslie PN, Green DJ (2015). Impact of sympathetic nervous system activity on post-exercise flow-mediated dilatation in humans. J Physiol.

[CR7] Birk GK, Dawson EA, Atkinson C, Haynes A, Cable NT, Thijssen DH, Green DJ (2012). Brachial artery adaptation to lower limb exercise training: role of shear stress. J Appl Physiol.

[CR8] Birk GK, Dawson EA, Batterham AM, Atkinson G, Cable T, Thijssen DH, Green DJ (2013). Effects of exercise intensity on flow mediated dilation in healthy humans. Int J Sports Med.

[CR9] Carter HH, Dawson EA, Birk GK, Spence AL, Naylor LH, Cable NT, Thijssen DH, Green DJ (2013). Effect of SR manipulation on conduit artery dilation in humans. Hypertension.

[CR10] Corretti MC, Anderson TJ, Benjamin EJ, Celermajer D, Charbonneau F, Creager MA, Deanfield J, Drexler H, Gerhard-Herman M, Herrington D, Vallance P, Vita J, Vogel R, International Brachial Artery Reactivity Task, F (2002). Guidelines for the ultrasound assessment of endothelial-dependent flow-mediated vasodilation of the brachial artery: a report of the international brachial artery reactivity task force. J Am Coll Cardiol.

[CR11] Crandall CG, Wilson TE (2011). Human cardiovascular responses to passive heat stress. Compr Physiol.

[CR12] Cullen T, Clarke ND, Hill M, Menzies C, Pugh CJA, Steward CJ, Thake CD (2020). The health benefits of passive heating and aerobic exercise: to what extent do the mechanisms overlap?. J Appl Physiol.

[CR13] Dawson EA, Alkarmi A, Thijssen DH, Rathore S, Marsman DE, Cable NT, Wright DJ, Green DJ (2012). Low-flow mediated constriction is endothelium-dependent: effects of exercise training after radial artery catheterization. Circ Cardiovasc Interv.

[CR14] Dawson EA, Green DJ, Cable NT, Thijssen DH (2013). Effects of acute exercise on flow-mediated dilatation in healthy humans. J Appl Physiol.

[CR15] Elliott RO, Alsalahi S, Fisher JP (2018). Impact of acute dynamic exercise on radial artery low-flow mediated constriction in humans. Eur J Appl Physiol.

[CR16] Facer-Childs ER, Pake K, Lee VY, Lucas SJE, Balanos GM (2019). Diurnal variations in vascular endothelial vasodilation are influenced by chronotype in healthy humans. Front Physiol.

[CR17] Fletcher GF, Ades PA, Kligfield P, Arena R, Balady GJ, Bittner VA, Coke LA, Fleg JL, Forman DE, Gerber TC, Gulati M, Madan K, Rhodes J, Thompson PD, American Heart Association Exercise, C. R., Prevention Committee of the Council on Clinical Cardiology, C. O. N. P. A., Metabolism, C. O. C., Stroke, N., Council On, E. and Prevention (2013). Exercise standards for testing and training: a scientific statement from the American Heart Association. Circulation.

[CR18] Franklin BA, Thompson PD, Al-Zaiti SS, Albert CM, Hivert MF, Levine BD, Lobelo F, Madan K, Sharrief AZ, Eijsvogels TMH, American Heart Association Physical Activity Committee of the Council On, L., Cardiometabolic, H., Council On, C., Stroke, N., Council on Clinical, C. and Stroke, C (2020). Exercise-related acute cardiovascular events and potential deleterious adaptations following long-term exercise training: placing the risks into perspective-an update: a scientific statement from the American heart association. Circulation.

[CR19] Goodman JM, Burr JF, Banks L, Thomas SG (2016). The acute risks of exercise in apparently healthy adults and relevance for prevention of cardiovascular events. Can J Cardiol.

[CR20] Gori T, Dragoni S, Lisi M, Di Stolfo G, Sonnati S, Fineschi M, Parker JD (2008). Conduit artery constriction mediated by low flow a novel noninvasive method for the assessment of vascular function. J Am Coll Cardiol.

[CR21] Gori T, Muxel S, Damaske A, Radmacher MC, Fasola F, Schaefer S, Schulz A, Jabs A, Parker JD, Munzel T (2012). Endothelial function assessment: flow-mediated dilation and constriction provide different and complementary information on the presence of coronary artery disease. Eur Heart J.

[CR22] Green DJ, Hopman MT, Padilla J, Laughlin MH, Thijssen DH (2017). Vascular adaptation to exercise in humans: role of hemodynamic stimuli. Physiol Rev.

[CR23] Hambrecht R, Adams V, Erbs S, Linke A, Krankel N, Shu Y, Baither Y, Gielen S, Thiele H, Gummert JF, Mohr FW, Schuler G (2003). Regular physical activity improves endothelial function in patients with coronary artery disease by increasing phosphorylation of endothelial nitric oxide synthase. Circulation.

[CR24] Hashimoto M, Akishita M, Eto M, Ishikawa M, Kozaki K, Toba K, Sagara Y, Taketani Y, Orimo H, Ouchi Y (1995). Modulation of endothelium-dependent flow-mediated dilatation of the brachial artery by sex and menstrual cycle. Circulation.

[CR25] He GW, Taggart DP (2016). Spasm in arterial grafts in coronary artery bypass grafting surgery. Ann Thorac Surg.

[CR26] Hijmering ML, Stroes ES, Olijhoek J, Hutten BA, Blankestijn PJ, Rabelink TJ (2002). Sympathetic activation markedly reduces endothelium-dependent, flow-mediated vasodilation. J Am Coll Cardiol.

[CR27] Humphreys RE, Green DJ, Cable NT, Thijssen DH, Dawson EA (2014). Low-flow mediated constriction: the yin to FMD's yang?. Expert Rev Cardiovasc Ther.

[CR28] Hwang IC, Kim KH, Choi WS, Kim HJ, Im MS, Kim YJ, Kim SH, Kim MA, Sohn DW, Zo JH (2012). Impact of acute exercise on brachial artery flow-mediated dilatation in young healthy people. Cardiovasc Ultrasound.

[CR29] Johnson BD, Mather KJ, Newcomer SC, Mickleborough TD, Wallace JP (2012). Brachial artery flow-mediated dilation following exercise with augmented oscillatory and retrograde shear rate. Cardiovasc Ultrasound.

[CR30] Laughlin MH, Newcomer SC, Bender SB (2008). Importance of hemodynamic forces as signals for exercise-induced changes in endothelial cell phenotype. J Appl Physiol.

[CR31] Padilla J, Johnson BD, Newcomer SC, Wilhite DP, Mickleborough TD, Fly AD, Mather KJ, Wallace JP (2008). Normalization of flow-mediated dilation to shear stress area under the curve eliminates the impact of variable hyperemic stimulus. Cardiovasc Ultrasound.

[CR32] Padilla J, Young CN, Simmons GH, Deo SH, Newcomer SC, Sullivan JP, Laughlin MH, Fadel PJ (2010). Increased muscle sympathetic nerve activity acutely alters conduit artery shear rate patterns. Am J Physiol Heart Circ Physiol.

[CR33] Padilla J, Simmons GH, Vianna LC, Davis MJ, Laughlin MH, Fadel PJ (2011). Brachial artery vasodilatation during prolonged lower limb exercise: role of shear rate. Exp Physiol.

[CR34] Razminia M, Trivedi A, Molnar J, Elbzour M, Guerrero M, Salem Y, Ahmed A, Khosla S, Lubell DL (2004). Validation of a new formula for mean arterial pressure calculation: the new formula is superior to the standard formula. Catheter Cardiovasc Interv.

[CR35] Song SW, Sul SY, Lee HJ, Yoo KJ (2012). Comparison of the radial artery and saphenous vein as composite grafts in off-pump coronary artery bypass grafting in elderly patients: a randomized controlled trial. Korean Circ J.

[CR36] Takase B, Uehata A, Akima T, Nagai T, Nishioka T, Hamabe A, Satomura K, Ohsuzu F, Kurita A (1998) Endothelium-dependent flow-mediated vasodilation in coronary and brachial arteries in suspected coronary artery disease. Am J Cardiol 82:1535–9, A7-8. 10.1016/s0002-9149(98)00702-4 10.1016/s0002-9149(98)00702-4 9874063

[CR37] Tanaka H, Monahan KD, Seals DR (2001). Age-predicted maximal heart rate revisited. J Am Coll Cardiol.

[CR38] Thijssen DH, Dawson EA, Tinken TM, Cable NT, Green DJ (2009). Retrograde flow and shear rate acutely impair endothelial function in humans. Hypertension.

[CR39] Thijssen DH, Atkinson CL, Ono K, Sprung VS, Spence AL, Pugh CJ, Green DJ (2014). Sympathetic nervous system activation, arterial shear rate, and flow-mediated dilation. J Appl Physiol.

[CR40] Thijssen DHJ, Bruno RM, Van Mil A, Holder SM, Faita F, Greyling A, Zock PL, Taddei S, Deanfield JE, Luscher T, Green DJ, Ghiadoni L (2019). Expert consensus and evidence-based recommendations for the assessment of flow-mediated dilation in humans. Eur Heart J.

[CR41] Thompson PD, Franklin BA, Balady GJ, Blair SN, Corrado D, Estes NA, Fulton JE, Gordon NF, Haskell WL, Link MS, Maron BJ, Mittleman MA, Pelliccia A, Wenger NK, Willich SN, Costa F, Council AHA, on Nutrition, P. A., Metabolism, American Heart Association Council on Clinical, C. & American College of Sports, M. (2007). Exercise and acute cardiovascular events placing the risks into perspective: a scientific statement from the American heart association council on nutrition, physical activity, and metabolism and the council on clinical cardiology. Circulation.

[CR42] Tinken TM, Thijssen DH, Hopkins N, Black MA, Dawson EA, Minson CT, Newcomer SC, Laughlin MH, Cable NT, Green DJ (2009). Impact of shear rate modulation on vascular function in humans. Hypertension.

[CR43] Tinken TM, Thijssen DH, Hopkins N, Dawson EA, Cable NT, Green DJ (2010). Shear stress mediates endothelial adaptations to exercise training in humans. Hypertension.

[CR44] Tremblay JC, Coombs GB, Howe CA, Vizcardo-Galindo GA, Figueroa-Mujica RJ, Bermudez D, Tymko MM, Villafuerte FC, Ainslie PN, Pyke KE (2019). Global Reach 2018: reduced flow-mediated dilation stimulated by sustained increases in shear stress in high-altitude excessive erythrocytosis. Am J Physiol Heart Circ Physiol.

[CR45] Vianna LC, Silva BM, Nobrega AC (2014). Sex differences in blood pressure responses to mental stress are abolished after a single bout of exercise: underlying hemodynamic mechanisms. J Physiol Sci.

[CR46] Watts K, Beye P, Siafarikas A, Davis EA, Jones TW, O’driscoll G, Green DJ (2004). Exercise training normalizes vascular dysfunction and improves central adiposity in obese adolescents. J Am Coll Cardiol.

[CR47] Weissgerber TL, Davies GA, Tschakovsky ME (2010). Low flow-mediated constriction occurs in the radial but not the brachial artery in healthy pregnant and nonpregnant women. J Appl Physiol.

[CR48] Ziegler T, Bouzourene K, Harrison VJ, Brunner HR, Hayoz D (1998). Influence of oscillatory and unidirectional flow environments on the expression of endothelin and nitric oxide synthase in cultured endothelial cells. Arterioscler Thromb Vasc Biol.

